# Ethical Leadership, Leader-Member Exchange and Feedback Seeking: A Double-Moderated Mediation Model of Emotional Intelligence and Work-Unit Structure

**DOI:** 10.3389/fpsyg.2017.01174

**Published:** 2017-07-11

**Authors:** Jing Qian, Bin Wang, Zhuo Han, Baihe Song

**Affiliations:** ^1^Department of Human Resource Management, Business School, Beijing Normal University Beijing, China; ^2^Beijing Key Laboratory of Applied Experimental Psychology, National Demonstration Center for Experimental Psychology Education, Beijing Normal University Beijing, China; ^3^Faculty of Psychology, Beijing Normal University Beijing, China

**Keywords:** ethical leadership, leader-member exchange, feedback seeking, emotional intelligence, work-unit structure

## Abstract

This research elucidates the role of ethical leadership in employee feedback seeking by examining how and when ethical leadership may exert a positive influence on feedback seeking. Using matched reports from 64 supervisors and 265 of their immediate employees from a hotel group located in a major city in China, we proposed and tested a moderated mediation model that examines leader-member exchange (LMX) as the mediator and emotional intelligence as well as work-unit structure as double moderators in the relationships between ethical leadership and followers’ feedback-seeking behavior from supervisors and coworkers. Our findings indicated that (1) LMX mediated the positive relationship between ethical leadership and feedback seeking from both ethical leaders and coworkers, and (2) emotional intelligence and work-unit structure served as joint moderators on the mediated positive relationship in such a way that the relationship was strongest when the emotional intelligence was high and work-unit structure was more of an organic structure rather than a mechanistic structure.

## Introduction

It has long been clear that people are reluctant to provide others with feedback; it is even more difficult for employees to gain certain resources through a formal human resource system in a timely fashion (Ashford et al., 2003; Anseel et al., 2015). Instead of passively waiting for feedback to be provided, employees may actively seek feedback to adapt and develop. Feedback seeking can generate a wide range of benefits for employees, from the question of “how am I doing” to survive and fit in (i.e., figuring out the “should self”) to the question of “am I on the right track to achieve the future goals” to develop and grow (i.e., figuring out the “possible self”). In light of this, scholars and practitioners increasingly take up the question of what factors can enhance or restrict such behavior. Comprehensive reviews (see the reviews: Levy et al., 1995; Ashford et al., 2003; Anseel et al., 2015) reveal that feedback seekers’ personal characteristics as well as contextual factors are antecedents of feedback seeking at work (e.g., Fedor et al., 1992; Callister et al., 1999; Wanberg and Kammeyer-Mueller, 2000; Steelman et al., 2004; Janssen and Prins, 2007).

Given the dominant role that leaders play in individual employees’ work lives (Chen et al., 2015), a few recent studies of feedback seeking attempted to demonstrate leaders’ impact on employees’ feedback-seeking (e.g., Qian et al., 2012, 2016; Anseel et al., 2015; Dahling et al., 2015). Among these studies, researchers have identified the effects of various types of positive leadership on feedback seeking, such as transformational leadership (Anseel et al., 2015), and authentic leadership (Qian et al., 2012). Following this research line, in this paper we focus on one of the most effective positive leadership styles, named ethical leadership. Ethical leadership has been defined as “the demonstration of normatively appropriate conduct through personal actions and interpersonal relationships, and the promotion of such conduct to followers through two-way communication, reinforcement, and decision-making” (Brown et al., 2005, p. 120). It could generate a range of positive outcomes (e.g., Mayer et al., 2009, 2012; Walumbwa et al., 2011b). It remains unclear, however, whether – and if so, how and why – ethical leadership could exert influence on employees’ feedback-seeking. A cursory look at the emergence of feedback-seeking behavior (Ashford, 1986; Anseel et al., 2015) reveals that having safe, fair, and trustworthy work norms is often placed in a central role, whereas evidence suggests that ethical leaders not only serve as the ethical role models with integrity, trustworthiness, and fairness but also exert transactional efforts to influence the climate as a whole (see the review by Ng and Feldman, 2014). Given the prevalence and far-reaching impact of ethical leadership, it is of both theoretical and empirical importance to investigate the influence of ethical leadership on employee feedback-seeking.

When explaining the effects of ethical leadership on employees’ feedback-seeking, we apply a social exchange model to explain the positive association between ethical leadership and feedback seeking. Social exchange theory is widely used to explain the influence of ethical leaders on their subordinates’ behaviors (Brown and Treviño, 2006). When subordinates under the management of ethical leaders receive ethical treatment, and feel their leaders’ trust in them, they are more likely to believe that they are in a high-quality social exchange relationship with their leaders (Brown et al., 2005). They tend to reciprocate by improving their performance (Walumbwa et al., 2011b). Leader-member exchange (LMX) is considered as an indicator of the quality of the social exchange relationship between supervisors and subordinates and has been examined as a mechanism to explain ethical leadership’s impact on employees’ performance (Walumbwa et al., 2011a,b). As such, we draw upon social exchange theory to propose that LMX serves as a mediator through which ethical leadership positively influences feedback seeking.

Recently, leadership research has called for an interactional approach to explicitly explore the contingent variables when examining the influence process of ethical leaders (Mayer et al., 2009; Eisenbeiss and van Knippenberg, 2015). Specifically, they suggest that it is important for researchers to further examine the contingencies under which ethical leadership may be related to work outcomes. More importantly, they call for future studies to examine both individual differences and contextual factors as potential moderators (e.g., Mayer et al., 2009). To address this issue, our moderated mediation model examines the joint moderating influence of an individual difference variable (i.e., emotional intelligence) as well as a work-unit characteristic variable (i.e., work-unit structure) on the impact of ethical leadership.

Emotional intelligence refers to the subset of social intelligence that involves the ability to perceive, assimilate, and understand one’s own and others’ feelings and emotions, and to use this information to manage emotions and guide thinking and actions in a certain context (Salovey and Mayer, 1989; Jordan et al., 2002). Asking for self-related information, no matter its nature (positive, negative, or neutral), can be emotionally charged (e.g., Ashford et al., 2003). Because feedback seeking is not a one-time event, research suggests that emotion-related abilities are essential for repeated feedback seeking (Brotheridge and Grandey, 2002). It is surprising, however, that little is known about the role of individuals’ differences in the ability to perceive, appraise, and regulate emotions influencing feedback seeking at work (Ashford and Cummings, 1983; Valcea et al., 2011). Indeed, Ashford et al. (2003) call for more studies to address the issue of emotional components in the feedback-seeking process. In this study, we address this gap by examining emotional intelligence as a moderator for the ethical leadership, LMX, and feedback-seeking relationships.

**FIGURE 1:**
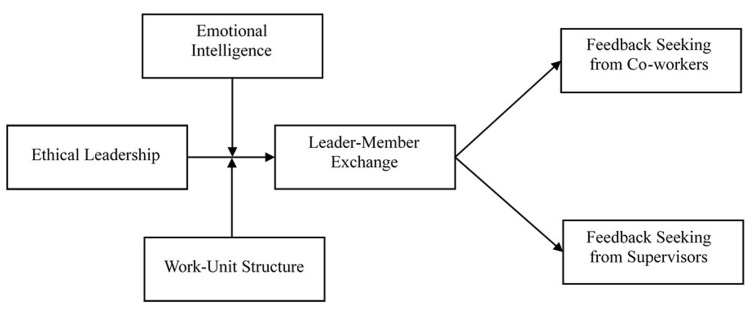
Hypothesized model.

Compared to studies examining individual difference moderators, less attention has paid to identify and empirically test contextual moderators on ethical leadership influence (Treviño et al., 2003; Brown et al., 2005; Brown and Treviño, 2006; Mayer et al., 2009). Work-unit structure refers to how much employee roles are defined by routines, the degree to which decision-making authority is concentrated at the top of the hierarchical structure, and the extent to which employees control the workflow (Dragoni and Kuenzi, 2012). It can range from formalized, centralized structures characterized by more certainty and stability (i.e., mechanistic structure) to a lack of formally defined tasks, decentralized structures characterized by more ambiguity and change (i.e., organic structure) (Dragoni and Kuenzi, 2012). We propose a moderation effect of work-unit structure on the mediation effect of ethical leadership because work-unit structure can determine the degree to which followers are willing to appreciate ethical leadership and further motivate them to process the relational benefits of ethical leadership to seek feedback at work. **Figure 1** serves as a guide for specifying our model.

### Ethical Leadership and Feedback Seeking

Ethical leadership researchers propose that ethical leadership behaviors are composed of two building blocks: being a moral person and being a moral manager (Treviño et al., 2000, 2003; Brown et al., 2005). The moral person aspect of ethical leadership suggests that ethical leaders have such characteristics as being honest, fair, and trustworthy (Treviño et al., 2000, 2003). And the moral manager aspect of ethical leadership includes the managerial efforts such as communication, rewards, and punishments to influence the desired ethical behaviors of their followers (Treviño et al., 2000). As moral persons, ethical leaders may serve as credible and trustworthy feedback sources. They convey a strong message of integrity, transparency, openness, fairness, trust, and respect for others (Treviño et al., 2000, 2003). On a daily basis, ethical leaders genuinely interact with subordinates and are capable of building and maintaining a higher-quality and credible two-way feedback channel. Employees of such leaders are likely to feel psychologically safe and seize the opportunity to seek feedback at work without any anxiety or stress (Walumbwa and Schaubroeck, 2009). Thus, employees are more willing to seek feedback from their ethical leaders. As moral managers, ethical leaders tend to exert managerial efforts and act as ethical role models to develop followers’ ethical behaviors (Brown and Treviño, 2006). They focus on both the outcome and the process, care about the people, and encourage employees to develop and grow ethically. As such, ethical leaders form an ethical climate and have the potential to create a work environment that supports learning and development while emphasizing the importance of being honest, fair, and trustworthy (Shin, 2012; Lu and Lin, 2014). In this context, subordinates are more likely to regard coworkers as a critical feedback source and proactively seek feedback from them to obtain the goals of learning and development (Qian et al., 2015; Ashford et al., 2016).

Hypothesis 1a: Ethical leadership is positively related to feedback-seeking behavior from supervisors.

Hypothesis 1b: Ethical leadership is positively related to feedback-seeking behavior from coworkers.

### Ethical Leadership, Leader-Member Exchange, and Feedback Seeking

Scholars have suggested that leaders in high LMX relationships tend to expect their followers to fulfill the reciprocity obligations for long-term personal development and collective interests instead of short-term personal gratification (e.g., Wang et al., 2005). In the present paper, we examine both leaders and coworkers as potential feedback sources. Although the majority of empirical studies have chosen immediate supervisors as the dominant source for employee feedback-seeking (e.g., VandeWalle et al., 2000; Chen et al., 2007), research found that both sources are of great importance and are not replaceable (Ashford et al., 2003). Thus, we draw upon social exchange theory to propose that LMX motivates employees to seek feedback from supervisors and coworkers.

As a social exchange relationship between employees and supervisors, LMX has received considerable attention in the organizational sciences (Masterson et al., 2000; Nahrgang et al., 2009; Walumbwa et al., 2011a). According to social exchange theory, high-quality LMX is considered as rewards or benefits from the supervisor for the employees. This could create obligations for the employees to reciprocate in an equivalent positive gesture to maintain the high-quality LMX (Blau, 1964; Emerson, 1976; Cropanzano and Mitchell, 2005; Chen et al., 2007). Since feedback information involves supervisors’ requirements and expectations (Ilgen et al., 1979; Judge et al., 2001; Chen et al., 2007; Lam et al., 2007; De Stobbeleir et al., 2011), after perceiving high LMX as beneficial acts from the supervisor, those employees in high-quality LMX in return are more likely to regard feedback-seeking as a way to clarify supervisors’ expectations in order to achieve goals of excellence and distinction, ultimately meeting ethical leaders’ expectations. Here, seeking feedback could be seen as exchange currency to help employees reciprocate. This may have a positive influence on the amount of effort that subordinates are willing to exert on feedback-seeking behavior. To maximize the potential benefits of feedback-seeking in reciprocating leaders, subordinates may try their best to seek more feedback information from various sources (both supervisors and coworkers). Therefore, there is a positive association between LMX and employees’ feedback-seeking behavior from supervisors and coworkers.

As moral persons and principled decision-makers who are honest and trustworthy, ethical leaders care about the greater merits of employees, the organization, and society (Treviño et al., 2003; Brown et al., 2005; Brown and Treviño, 2006). They practice what they preach and pay attention to establishing high-quality relationships with their subordinates, thus creating a strong supervisor feedback environment. This can be a good start. That is to say, when employees perceive fair treatments and considerations from their leaders, they are more likely to build emotional connections and mutual support mechanisms with their leaders and engage in high-quality social exchange relationships with their leaders as well (Wayne et al., 2002; Erdogan et al., 2006; Walumbwa et al., 2011b). This in turn could encourage employees to practice their feedback-seeking behavior with supervisors as a way to reciprocate their supervisors’ LMX and ethical leadership.

In addition, subordinates under the management of ethical leaders may reach a consensus that feedback-seeking is a useful and valuable method to reciprocate high-quality LMX with their supervisors. As a result, they may be more willing to engage in feedback-seeking from each other, fostering a strong coworker feedback environment. Previous studies suggest that supervisor and coworker feedback environments are positively related to subordinates’ tendency to seek feedback from these sources (Steelman et al., 2004; Ashford et al., 2016). Therefore, ethical leadership is likely to be associated with employees’ feedback-seeking behavior with supervisors and coworkers through establishing high-quality LMX. Accordingly,

*Hypothesis 2a*: *LMX is positively related to employees’ feedback-seeking behavior from supervisors.*

*Hypothesis 2b*: *LMX is positively related to employees’ feedback-seeking behavior from coworkers.*

*Hypothesis 3a*: *LMX mediates the relationship between ethical leadership and feedback-seeking behavior from supervisors.*

*Hypothesis 3b*: *LMX mediates the relationship between ethical leadership and feedback-seeking behavior from coworkers.*

### Ethical Leadership, Leader-Member Exchange, Emotional Intelligence, Work-Unit Structure and Feedback Seeking

Emotional intelligence captures individual differences in emotion-related abilities and plays a critical role in daily work life (Goleman, 1995; Brackett et al., 2011). We expect that emotional intelligence may moderate the mediated relationship between ethical leadership and employees’ feedback-seeking.

Employees with this high emotional intelligence are more aware of their and others’ feelings (Farh et al., 2012) and, more importantly, they are able to understand emotional information, know the relationships among them, and manage feelings accurately (Joseph and Newman, 2010). Dealing directly with emotional content, emotionally intelligent employees are able to address the unstated needs of their supervisors (Wolff et al., 2002). With this ability, they can easily strengthen the emotional connections with their supervisors and, thus, enhance the impact of ethical leadership as well as LMX. In addition, high emotionally intelligent employees are able to use emotions to facilitate thinking, tend to anticipate negative emotions with positive information (Jordan et al., 2002), and focus more on actions (Law et al., 2004; Van Rooy and Viswesvaran, 2004). Given that the ethical leaders’ influence on LMX and feedback seeking is not a one-time event, these emotional abilities could direct employees to the information, the value behind it, and the follow-up actions without paying too much attention on the possible negative side of this emotion-charged process (Ashford et al., 2003). Thus, this mediation relationship is stronger for employees with high emotional intelligence. In contrast, employees with low emotional intelligence cannot accurately observe and recognize supervisors’ emotions when they interact with their leaders. They may fail to appropriately respond. As a result, their interactions with leaders are managed in a less effective manner. In addition, those who possess low emotional intelligence are considered to be more aggressive and are more likely to engage in conflictual behaviors (Brackett et al., 2011). These behaviors could impair their relationships with ethical leaders, and the quality of their relationships may be lower. Therefore, the positive influence of ethical leadership on enhancing high-quality LMX is less effective for those with low emotional intelligence.

Work-unit structure shapes the type and extent of communication flow within the organization (Dragoni and Kuenzi, 2012). According to Hage (1965) and van de Ven (1976), the properties of work-unit structure (e.g., its size, organizational level, centralization, and complexity) have a direct influence on employee attitudes and behaviors. Given the potential moderating effect of contextual factors on ethical leadership’s influence (e.g., Blumberg and Pringle, 1982; Bandura, 1986), we suggest the magnitude of the indirect effect of ethical leadership on employees’ feedback-seeking may be moderated by work-unit structure.

Work-unit structure may determine the degree to which followers are willing to appreciate ethical leadership and its influence. In mechanistic structures, there is a high level of formalization and centralization of control and authority (Weber, 1947). They clearly define how much work should be finished, how to complete it, and what the deadline is (Dragoni and Kuenzi, 2012). The features of mechanistic structures also exacerbate the power distance between leaders and employees, which may hinder the ethical leadership to exert positive impact on leader-member exchange and employees’ feedback-seeking. Different from mechanistic organizations, organic structures involve decentralization of control and authority, and few formal procedures (Burns and Stalker, 1966). It relies more on interpersonal transactions (Ambrose and Schminke, 2003), such as face-to-face communication (Lengel and Daft, 1987), informal control (Ouchi, 1980), and interpersonal interactions (Nadler and Tushman, 1990). These features promote the exchange relationship between leaders and employees, resulting in a higher level of employees’ initiative and discretion to perform the work better. Additionally, the greater communization and lower level of power distance provide greater opportunity for the ethical leadership to exert a greater influence on the employees’ feedback-seeking. Accordingly,

Hypothesis 4: Emotional intelligence and work-unit structure simultaneously moderate the mediated effects of LMX on the positive relation between ethical leadership and subordinate feedback seeking from supervisors and coworkers in the way that the relationship will be the strongest when emotional intelligence is high and work-unit structure is an organic structure rather than a mechanistic structure.

## Materials and Methods

### Participants and Procedure

Participants in the current study were employees from a hotel group located in a major city in Northern China. Separate questionnaires were designed for supervisors and subordinates to minimize potential common-method biases. The supervisor questionnaires were distributed to 70 supervisors at company meetings held for supervisors, and the subordinate questionnaires were distributed to 350 immediate subordinates of these supervisors. The surveys took place over the course of four meetings in 1 month, all of which were designed for training purposes to be limited in size. The participants from each meeting were randomly selected by computer. With the assistance of human resource management, five immediate subordinates for each supervisor were randomly selected by computer. Each questionnaire was assigned an identification number so that the supervisor ratings could be matched with the subordinate responses. Participants were asked to fill out the questionnaires, seal them in envelopes, and return the questionnaires 2 weeks later to a box placed outside a company meeting venue. Two short messages were sent to the participants 3 days after the questionnaires were distributed and 1 day before the company meeting to encourage participants to complete the survey and to remind them to return the surveys. In total, 64 supervisors and 281 subordinate questionnaires were returned, with response rates of 91.43 and 80.28%, respectively. After eliminating the uncompleted and unmatched questionnaires, the final sample included a total of 265 pairs (64 supervisors and 265 subordinates), constituting the sample used for the current study. Of the 265 subordinates, 58.6% were male (*SD* = 0.48). They reported an average age of 32.69 years (*SD* = 8.25), an average organizational tenure of 6.24 years (*SD* = 3.95), and an average of 11.12 years of education (*SD* = 3.13). Of the 64 supervisors, 81% were male, the average reported age was 33.32 years (*SD* = 7.2), and the average reported organizational tenure was 7.64 years (*SD* = 4.36).

### Measures

#### Ethical Leadership

We measured supervisors’ ethical leadership with a 10-item scale developed by Brown et al. (2005). Example items are “my supervisor talks about the importance of ethics” and “my supervisor sets an example of how to do things the right way in terms of ethics.” Response options ranged from 1, “strongly disagree” to 7, “strongly agree.” The scale’s reliability was 0.73.

#### LMX

We used the seven-item LMX-7 scale developed by Scandura and Graen (1984). A sample item is “How well do you feel that your immediate supervisor understands your problems and needs?” Response options ranged from 1, “strongly disagree” to 7, “strongly agree.” The scale’s reliability was 0.81.

#### Feedback Seeking from Supervisor

Feedback seeking from supervisors was measured with a five-item scale validated by VandeWalle et al. (2000). Each supervisor was asked to provide his or her own ratings of how often each of the five aspects of feedback (i.e., adequacy of job performance, technical aspects of the job, the values and attitudes of the firm, role expectations, and social behaviors) was sought by the rated subordinate. Sample items included: “How often does this subordinate ask you for feedback about his or her overall job performance?” and “How often does this subordinate ask you for feedback about his social behaviors?” Response options ranged from 1, “never” to 7, “always.” The scale’s reliability was 0.71.

#### Feedback Seeking from Coworker

Measured with a five-item scale validated by VandeWalle et al. (2000), each employee participant provided his or her own ratings of how frequently they asked their coworkers for each of the five aspects of feedback (i.e., the inadequacies of overall job performance, technical aspects of the job, values and attitudes of the firm, role expectations, and social behaviors). Their scores were averaged to rate feedback seeking from immediate coworkers. Response options ranged from 1, “never,” to 7, “always.” Response options ranged from 1, “never” to 7, “always.” The alpha reliability for the scale was 0.74.

#### Emotional Intelligence

We used the sixteen-item WLEIS (Wong and Law Emotional Intelligence Scale) developed by Wong and Law (2002), each employee participant provided his or her own ratings of emotional intelligence. This scale measures the four dimensions of emotional intelligence: self-emotion appraisal and expression, other-emotion appraisal and recognition, self-emotion regulation, and self-use of emotions to increase performance (Davies et al., 1998). Each dimension was indicated by four-items. Sample items are “I have good understanding of my own emotions,” “I am a good observer of others’ emotions,” “I am able to control my temper and handle difficulties rationally,” and “I would always encourage myself to try my best.” Response options ranged from 1, “strongly disagree” to 7, “strongly agree.” The alpha reliability for the scale was 0.91.

#### Work-Unit Structure

We assessed work-unit structure by using the seven-item scale from Khandwalla (1976, 1977). Each employee was asked to rate the extent to which paired statements describe the structure of his or her work unit on a seven-point scale. Response options ranged from 1, “strongly disagree” to 7, “strongly agree.” Sample items are “Highly structured channels of communication and highly restricted access to important operating information” vs. “Open channels of communication with important financial and operating information flowing quite freely throughout the organization.” The alpha reliability for the scale was 0.71.

#### Control Variables

Previous research on LMX included participants’ gender, education, and organization tenure as control variables (e.g., Gu et al., 2015). A recent review of feedback-seeking also noted the potential effects of demographic variables (e.g., age, gender, race, and organizational tenure, etc.) on the feedback-seeking process (Ashford et al., 2016). Accordingly, we controlled for the participants’ age, gender, education, and company tenure. Age, education, and tenure were measured by the number of years. Gender was coded 0 for “female” and 1 for “male.”

## Results

### Analytic Plan

First, preliminary analyses using Pearson’s correlational tests and independent samples *t*-tests examined the zero-order correlations among study variables as well as whether they differed on any demographic variables (i.e., age, years of tenure, gender, and educational level). Next, procedures recommended by Preacher and Hayes (2004) were performed to test for mediation. One thousand bootstrapping resamples generated 95% confidence intervals to determine if there was an indirect effect of leader-member exchange on the relations between ethical leadership and two types of feedback-seeking behaviors (i.e., from coworkers and from supervisors). When significant mediation was established, conditional indirect effect procedures recommended by Hayes and Preacher (2014) were performed to estimate if the mediation was conditional depending on the levels of two theoretically proposed moderators (i.e., emotional intelligence and work-unit structure). One thousand bootstrapping resamples generated 95% confidence intervals was generated and two double-moderated mediation models (Preacher et al., 2007) were tested to determine if the conditional mediation model was significant for feedback-seeking behaviors from supervisors and for feedback-seeking behaviors from coworkers.

### Preliminary Analysis

Descriptive statistics and zero-order correlations among study variables are shown on **Table 1**. Ethical leadership was significantly and positively related with leader-member exchange (*r* = 0.24; *p* < 0.000) and with feedback-seeking behaviors from supervisors (*r* = 0.15; *p* = 0.013). It was also moderately related to feedback-seeking behaviors from co-workers (*r* = 0.11; *p* = 0.08). Additionally, leader-member exchange was significantly and positively correlated with feedback-seeking behaviors from supervisors (*r* = 0.23; *p* < 0.000) and with that from co-workers (*r* = 0.23; *p* < 0.000). Additional Pearson’s correlational tests indicated that participants’ age, years of tenure, and educational levels were not significantly associated with any study variables. A series of independent samples *t*-tests showed that participant gender had no significant effect on any study variables.

**Table 1:** Means, standard deviation, and zero-order correlations among study variables.

Variable	*M*	*SD*	1	2	3	4	5
(1) Ethical leadership	5.63	0.85					
(2) Leader-member exchange	5.63	0.67	0.24^∗∗∗^				
(3) Feedback seeking from co-workers	5.14	0.78	0.11^+^	0.23^∗∗∗^			
(4) Feedback seeking from supervisors	5.73	0.80	-0.15^∗∗^	0.23^∗∗∗^	0.43^∗∗∗^		
(5) Emotional intelligence	4.19	0.56	0.08	0.02	-0.03	-0.01	
(6) Work-unit structure	5.78	0.60	-0.05	0.07	-0.03	-0.00	0.12

^+^*p < 0.1, ^∗∗^*p* < 0.01, ^∗∗∗^*p* < 0.001*.

### Mediational Analysis

The first mediational model examined whether leader-member exchange mediated the relations between ethical leadership and feedback-seeking behavior from coworkers. We applied the non-parametric resampling procedure to assess mediation with SPSS INDIRECT Macros as recommended by Preacher and Hayes (2004). This bootstrapping technique was considered the most powerful and reasonable to obtain confidence intervals for indirect effects (Williams and Mackinnon, 2008). Mediation was established if the indirect effect was significant and the confidence intervals did not contain zero. The results showed that ethical leadership was related to more leader-member exchange, which in turn was related to more feedback-seeking behaviors from coworkers (indirect effects point estimate = 0.0876, *SE* = 0.09, 95% BCa CI = 0.0228 - 0.1812). Similarly, ethical leadership was related to more leader-member exchange, which in turn was related to more feedback-seeking behaviors from supervisors (indirect effect point estimate = 0.0842, *SE* = 0.03, 95% BCa CI = 0.0289 - 0.1838).

### Double-Moderated Mediation Analysis

**FIGURE 2:**
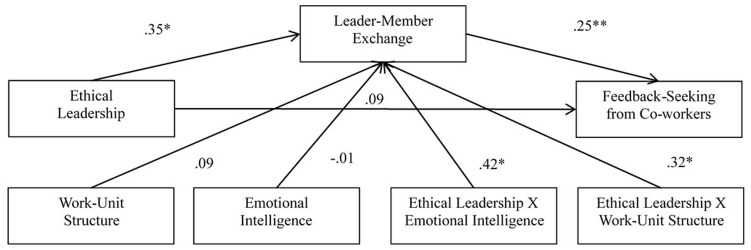
Results of structural equation modeling.

Based on significant mediation models and theoretical consideration, we then applied SPSS PROCESS Macros Model 9 to examine whether, how, and under what conditions a given effect occurs depending on two moderators (emotional intelligence and work-unit structures). The overall testing models were presented at **Figure 1**. The first double-moderated mediation tested PROCESS Model 9 with feedback-seeking behaviors from coworkers as the outcome variable. All path coefficients are demonstrated in **Figure 1** and the specific indirect effect and standard errors at different values of emotional intelligence and work-unit structure are presented in **Table 2**. As outlined by Hayes (2013), the conditional indirect effect was demonstrated if the interactions between independent variable and moderators were significant and the bootstrapping confidence intervals did not contain zero. From data shown on **Figure 2** and in **Table 2**, it seemed that emotional intelligence and work-unit structure significantly moderated the indirect effect of ethical leadership on feedback seeking from coworkers via leader-member exchange. Specifically, from lower to higher levels on both moderators, the interactional effect between ethical leadership and emotional intelligence as well as that between ethical leadership and work-unit structure were increasing. Their effect on leader-member exchange was strongest when both emotional intelligence and work-unit structures were at their highest levels (i.e., organic structure), which in turn led to greater values of feedback-seeking behaviors from coworkers.

**Table 2:** Double conditional indirect effect of ethical leadership on feedback seeking (FB-C, feedback-seeking from co-workers; FB-S, feedback-seeking from supervisors) at specific levels of emotional intelligence (EI) and work-unit structure (WU).

Variable						BC 1000 BOOT			
EI	WU	IND		*SE*		LL95		UL95	
		FB-C	FB-S	FB-C	FB-S	FB-C	FB-S	FB-C	FB-S
LOW	LOW	-0.02	-0.02	0.06	0.06	-0.1237	-0.1434	0.1368	0.1069
LOW	MEAN	0.03	0.03	0.03	0.05	-0.0514	-0.0534	0.1639	0.1478
LOW	HIGH	0.08	0.07	0.07	0.06	-0.0519	-0.0407	0.2337	0.2143
MEAN	LOW	0.03	0.04	0.05	0.05	-0.0447	-0.0502	0.1610	0.1689
MEAN	MEAN	0.09ˆ*	0.08ˆ*	0.04	0.04	0.0311	0.0309	0.1949	0.1751
MEAN	HIGH	0.14ˆ*	0.13ˆ*	0.06	0.06	0.0441	0.0439	0.2862	0.2924
HIGH	LOW	0.10	0.10	0.07	0.08	-0.0183	-0.0244	0.2757	0.2850
HIGH	MEAN	0.15ˆ*	0.14ˆ*	0.06	0.07	0.0529	0.0326	0.2956	0.3174
HIGH	HIGH	0.20ˆ*	0.19ˆ*	0.07	0.08	0.0769	0.0618	0.3742	0.4121

*Coefficients represent specific indirect effects and standard errors at different values of both moderators, *EI* and *WU*, and lower and upper bound of 95% BC bootstrap confidence interval for that effect using 1,000 bootstrap samples. LOW signifies values at one standard deviation below the mean; MEAN signifies values at the mean; HIGH signifies values at one standard deviation above the mean. ^∗^*p* < 0.05*.

**FIGURE 3:**
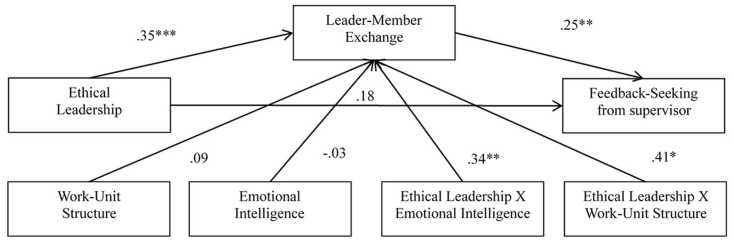
Results of structural equation modeling.

The second double-moderated mediation tested PROCESS Model 9 with feedback-seeking behaviors from supervisors as the outcome variable. All path coefficients are shown in **Figure 3** and the specific indirect effect and standard errors at different values of emotional intelligence and work-unit structure are presented in **Table 2**. The results were similar to that with feedback seeking from coworkers as the outcome variable, that emotional intelligence and work-unit structure significantly moderated the indirect effect of ethical leadership on feedback seeking from supervisors via leader-member exchange. From lower to higher levels of emotional intelligence and work-unit structure, the interactional effect between ethical leadership and emotional intelligence as well as that between ethical leadership and work-unit structure was increasing. Their effect was strongest when both emotional intelligence and work-unit structures were at their highest levels (i.e., organic structure), which in turn led to greater values of feedback-seeking behaviors from supervisors.

## Discussion

In this study, we examined the role of ethical leadership on promoting employees’ feedback-seeking behavior. The results showed that LMX mediated the positive relationship between ethical leadership and feedback seeking from both ethical leaders and coworkers. Additionally, we found that emotional intelligence and work-unit structure served as joint moderators on the mediated positive relationship in a way that the relationship was strongest when the emotional intelligence was high and work-unit structure was an organic structure rather than a mechanistic structure.

### Theoretical Implications

Our research contributes to the literature of ethical leadership and feedback seeking in several ways. First, our results showed that employees are encouraged to seek feedback from both supervisors and coworkers when leaders impose ethical leadership, which filled the gap of feedback-seeking literature. Although previous researchers have found that leadership style emerged to significantly influence employee feedback-seeking, they mainly focus on transformational and transactional leadership theory (Madzar, 2001; Levy et al., 2002), and authentic leadership theory (Qian et al., 2012), little is known about the impact of ethical leadership. Another lack of insight concerns feedback sources. Because previous studies mainly examined feedback seeking from supervisors (e.g., Ashford et al., 2003, 2016), we addressed this shortcoming by proposing that employees seek feedback from both supervisors and coworkers under ethical leadership.

Second, we provided a new perspective to explain why and how leadership behaviors could exert influence on employees’ feedback-seeking beyond the traditional cost and value mechanisms (e.g., Chun et al., 2014). While LMX has been universally investigated as a mediator between leadership and their outcomes (e.g., Wang et al., 2005; Chan and Mak, 2012; Decoster et al., 2014; Peng et al., 2014; Gu et al., 2015), we are the first to employ LMX theory to open the black box of the positive relationship between ethical leadership and feedback seeking. We encourage future researchers to examine other potential mechanisms through which leaders could exert influence on employees’ feedback-seeking.

Third, we responded to the recent call for an interactional approach to explore the possible contingent variables which may moderate the process of ethical leadership (Mayer et al., 2009; Eisenbeiss and van Knippenberg, 2015) by examining the joint moderating effect of emotional intelligence and work-unit structure on the mediated positive relationship between ethical leadership and feedback-seeking behavior. Primarily, we hypothesized and found the joint moderating effect of emotional intelligence and work-unit structure on the mediated relationship. However, work is needed to investigate other individual differences such as feedback self-efficacy (e.g., Alicke and Sedikides, 2009) and contextual factors such as organizational support (e.g., Teunissen et al., 2009; Nifadkar et al., 2012).

### Practical Implications

Our findings have several practical implications. First, it is worthwhile to implement ethical leadership in managerial practice. Our study shows that ethical leadership could promote high-quality LMX, which enhances employee feedback seeking. Other than some antecedents of feedback seeking such as learning orientation and personality, ethical leadership can be developed through training programs (e.g., Brown et al., 2005). Organizations could, therefore, provide opportunities for supervisors to develop their ethical leadership to motivate employees to seek feedback. Second, our research suggests that ethical leadership is socially interactive and dynamic, which influences employee feedback-seeking through social exchange between leaders and followers. When leaders fail to meet followers’ reciprocity expectations or related requirements of a high-quality relationship (e.g., reciprocity, personal development, and social bonding; Dvir et al., 2002), the impact of ethical leadership on feedback seeking will be less effective. We, therefore, suggest organizations incorporate relationship-building training into ethical leadership development programs. Third, we suggest organizations provide employees with programs that enhance emotional intelligence while building organic structures. Our findings suggest that employees with higher emotional intelligence are more likely to be influenced by ethical leadership and that an organic structure encourages employees to seek feedback.

### Strengths and Limitations

The present study has several strengths. First, it takes an initial step toward understanding the influence of ethical leadership on feedback seeking as well as presenting a new angle for explaining the ethical leadership/feedback-seeking relationship. Drawing upon social exchange theory, we show how LMX plays a role in channeling the benefits of ethical leadership to promote feedback seeking at work. Second, with the present paper, we provide new conceptual insights and empirical evidence in the contingency studies about the influence of ethical leadership on feedback seeking. Unlike previous studies that examined either situational (Schaubroeck et al., 2012) or individual differences (Eisenbeiss and van Knippenberg, 2015) as boundary conditions, this study examines the joint moderating influence of an individual difference variable (i.e., emotional intelligence) and a work-unit characteristic variable (i.e., work-unit structure) on the influence process of ethical leadership. This could potentially provide a more complete understanding of the conditions under which ethical leadership influences employees’ feedback-seeking. Third, while past research has largely suggested that both supervisors and co-workers are important feedback sources, the majority of empirical studies have chosen immediate supervisors as the dominant source of employees’ feedback-seeking (e.g., VandeWalle et al., 2000; Chen et al., 2007). The present study thus adds value to the feedback-seeking literature by investigating whether ethical leadership could help encourage feedback seeking from both sources. Fourth, we have avoided collecting data from a single source by designing separate questionnaires for supervisors and subordinates, which could minimize potential common-method biases. Lastly, our study has employed a sample of subordinate and supervisor dyads from China. This is critical because feedback-seeking scholars have long raised the concern about whether feedback-seeking theories previously supported in Western societies might be generalized to other cultures (e.g., Robinson and Weldon, 1993; Bailey et al., 1997; Sully De Luque and Sommer, 2000). Thus, it is timely to assess our model in China. Together, these advances extend classic and contemporary discussions of ethical leadership as well as feedback-seeking behavior.

Inevitably, our study comes with some limitations. The first limitation concerns causality. Although our moderated mediation model is built upon solid theories, the cross-sectional design does not allow us to conclude definitively causal relations among study variables. Future studies could apply longitudinal research design or experiments. Second, the data used in our study is perceptual rather than behavioral. Specifically, participants rated the level of ethical leadership based on their subjective perceptions rather than independent measures that substantiate these perceptions. We recommend that future researchers collect behavioral data of ethical leadership with objective measures. Third, the generalizability of our findings is limited at present because our research used a sample of supervisors and employees from China. Future replication studies are warranted to ascertain the general applicability of our findings in other cultures.

## Conclusion

The present study suggests that leaders can set the ethical tone of an organization, which has significant influence on employee feedback-seeking through LMX. Meanwhile, our research indicates that high emotional intelligence and work-unit structure enhance the positive effect of ethical leadership on employee feedback-seeking.

## Ethics Statement

All procedures performed in studies involving human participants were in accordance with the ethical standards of the institutional and/or national research committee and with the 1964 Helsinki Declaration and its later amendments or comparable ethical standards with written informed consent from all subjects. This research was approved by the Human Research Ethics Committee (HREC) at Business School, Beijing Normal University.

## Author Contributions

JQ and BW substantially contributed to the conception, the design of the work as well as the preparation of the draft. ZH contributed to the analysis and interpretation of the data. BS reviewed it critically and gave important intellectual input.

## Conflict of Interest Statement

The authors declare that the research was conducted in the absence of any commercial or financial relationships that could be construed as a potential conflict of interest.
